# Integrative systematic review and transcriptomic -machine learning analysis of molecular signatures in metaplastic breast cancer

**DOI:** 10.3332/ecancer.2026.2119

**Published:** 2026-05-07

**Authors:** Joshua Agilinko, Sonam Patel, Jogitha Selvarajah, Nicholas Tekkis, Mathew Vithayathil, Suzette Samlalsingh

**Affiliations:** 1 The Elm Breast Unit, King George Hospital, Barking, Havering and Redbridge University Hospitals NHS Trust, Ilford, Essex IG3 8YB, UK; 2 Department of Surgery and Cancer, Imperial College London, Hammersmith Hospital Campus, London W12 0NN, UK

**Keywords:** metaplastic breast cancer, transcriptomics, gene expression, machine learning, molecular profiling, precision oncology

## Abstract

**Background:**

Metaplastic breast cancer (MpBC) is a rare and aggressive breast cancer subtype characterised by marked histological heterogeneity, therapeutic resistance and poor clinical outcomes. Despite increasing molecular research, existing evidence remains fragmented, heterogeneous and poorly integrated, limiting clinical translation and biomarker validation.

**Methods:**

We developed an integrative analytical framework combining systematic review, quantitative meta-analysis, transcriptomic profiling and interpretable machine learning to identify and prioritise molecular markers in MpBC. A Preferred Reporting Items for Systematic Reviews and Meta Analyses-guided systematic review was conducted across PubMed, arXiv and Semantic Scholar. Effect sizes were standardised to Cohen’s d and synthesised using a random-effects model. Transcriptomic analysis was performed on the GSE165407 dataset using DESeq2 in R (RStudio version 1.1.463), with differentially expressed genes cross-referenced against literature-derived biomarkers. Supervised models including a multi-layer perceptron and boosted random forest were applied, with performance evaluated using receiver operating characteristic analysis. Model interpretability was assessed using SHapley Additive exPlanations.

**Results:**

Eleven studies met inclusion criteria. Meta-analysis demonstrated low heterogeneity and a pooled effect size of d = 0.74 (95% CI 0.59–0.88), indicating a consistent moderate-to-large biomarker signal across studies. Pathway enrichment revealed convergence on PI3K/AKT/mTOR signalling, immune modulation and epithelial -mesenchymal transition. Transcriptomic profiling demonstrated concordance with literature-derived markers. The random forest model achieved strong classification performance (AUC = 0.91), with high specificity and minimal misclassification. SHapley Additive exPlanations analysis identified both canonical (PI3KCA, RPL39, EXO1) and non-canonical (CD55, LARGE2) contributors to model prediction.

**Conclusion:**

This study provides an integrated synthesis linking systematic evidence, transcriptomic validation and interpretable machine learning in MpBC. By reconciling fragmented literature with data-driven modelling, we identify a biologically coherent and clinically tractable molecular signature, offering a foundation for biomarker-driven stratification and translational validation.

## Introduction

Metaplastic breast cancer (MpBC) is a rare and highly aggressive subtype of breast carcinoma, accounting for approximately 0.2%–1% of all breast cancers [1]. It is characterised by pronounced phenotypic plasticity, defined by the coexistence of epithelial and mesenchymal components within the same tumour. The 2011 World Health Organisation classification recognises five principal histological subtypes, including squamous, spindle cell, low-grade adenosquamous and fibromatosis-like carcinomas, as well as MpBC with mesenchymal differentiation [2]. This marked histological heterogeneity reflects underlying biological complexity and contributes to diagnostic ambiguity, therapeutic resistance and poor clinical outcomes.

Although the majority of MpBCs fall within the triple-negative breast cancer (TNBC) spectrum, lacking expression of oestrogen receptor, progesterone receptor and HER2 [3], they represent a biologically distinct entity. Compared with conventional TNBC, MpBC exhibits enhanced chemoresistance, a greater propensity for early relapse and inferior survival outcomes. Current systemic therapies therefore remain suboptimal, and no molecularly stratified treatment paradigm has yet been established for this disease.

At the molecular level, MpBC is characterised by dysregulated programmes of epithelial–mesenchymal transition, stemness and immune modulation. Candidate drivers including HMGA2, RPL39 and TFAP2B have been implicated in tumour plasticity and aggressive phenotypes [4-6], while enrichment of stem cell-associated markers and immune checkpoint molecules suggests convergence on pathways of dedifferentiation and immune evasion [7,8]. Despite these advances, the current evidence base remains fragmented. Existing studies are frequently limited by small sample sizes, methodological heterogeneity and lack of independent validation, thereby constraining reproducibility and clinical translation [9].

A major unmet need in MpBC is the identification of robust, reproducible molecular signatures that can inform diagnosis, prognostication and therapeutic targeting. To date, most studies have evaluated biomarkers in isolation, without integration across datasets or analytical modalities, limiting their translational applicability. Given the rarity of MpBC and the consequent scarcity of large cohorts, approaches that maximise information integration are particularly valuable.

In this study, we implement an integrative analytical framework that combines systematic evidence synthesis, quantitative meta-analysis, transcriptomic profiling and interpretable machine learning to identify and prioritise molecular markers in MpBC. By reconciling heterogeneous findings across the literature with data-driven validation and model-based interpretation, this approach aims to resolve inconsistencies in biomarker discovery and identify clinically tractable molecular signatures with potential diagnostic and prognostic utility.

## Methodology

### Search strategy and study selection

A systematic review was conducted to identify studies reporting molecular markers in metaplastic breast cancer (MpBC) from database inception to June 2025. The review adhered to the Preferred Reporting Items for Systematic Reviews and Meta-Analyses (PRISMA) guidelines [10].

Studies were retrieved from PubMed, arXiv and Semantic Scholar to ensure comprehensive coverage of both peer-reviewed biomedical literature and emerging computational research. PubMed provided access to clinically curated studies, while arXiv and Semantic Scholar enabled inclusion of high-quality bioinformatic and machine learning studies.

Search strategies incorporated Medical Subject Headings (MeSH), Boolean operators and free-text terms to maximise sensitivity and specificity. Queries combined biomarker-related terms, including ‘Molecular Biomarkers’, ‘Genetic Markers’ and ‘Tumour Markers, Biological’, with MpBC-specific terminology such as ‘Metaplastic Breast Cancer’ and ‘Metaplastic Breast Neoplasms’. Additional filters were applied to capture diagnostic, prognostic and predictive studies.

To enhance screening efficiency and minimise selection bias, natural language processing approaches were integrated into the review workflow. Query expansion was guided by Population–Intervention–Comparator–Outcome (PICO) frameworks. Abstract classification was performed using rule-based text mining, regular expressions and named entity recognition implemented via spaCy and Bidirectional Encoder Representations from Transformers, enabling automated categorisation of studies against predefined inclusion criteria.

Reference lists of eligible studies were manually screened to identify additional relevant publications. Only human studies reporting primary molecular data in MpBC were included. Reviews, conference abstracts and studies limited to cell line or animal models were excluded.

### Eligibility criteria

Studies were included if they:

- reported genomic, transcriptomic or proteomic markers in MpBC
- provided quantitative outputs, including hazard ratios, odds ratios or differential expression analyses
- were published in peer-reviewed journals or high-quality indexed preprint repositories
- were available in full text with sufficient methodological detail to enable critical appraisal and data extraction [11]

Studies were excluded if they lacked primary data, were non-analytical in design or did not employ methodologies consistent with molecular biomarker evaluation.

### Study selection

**Figure 2.**
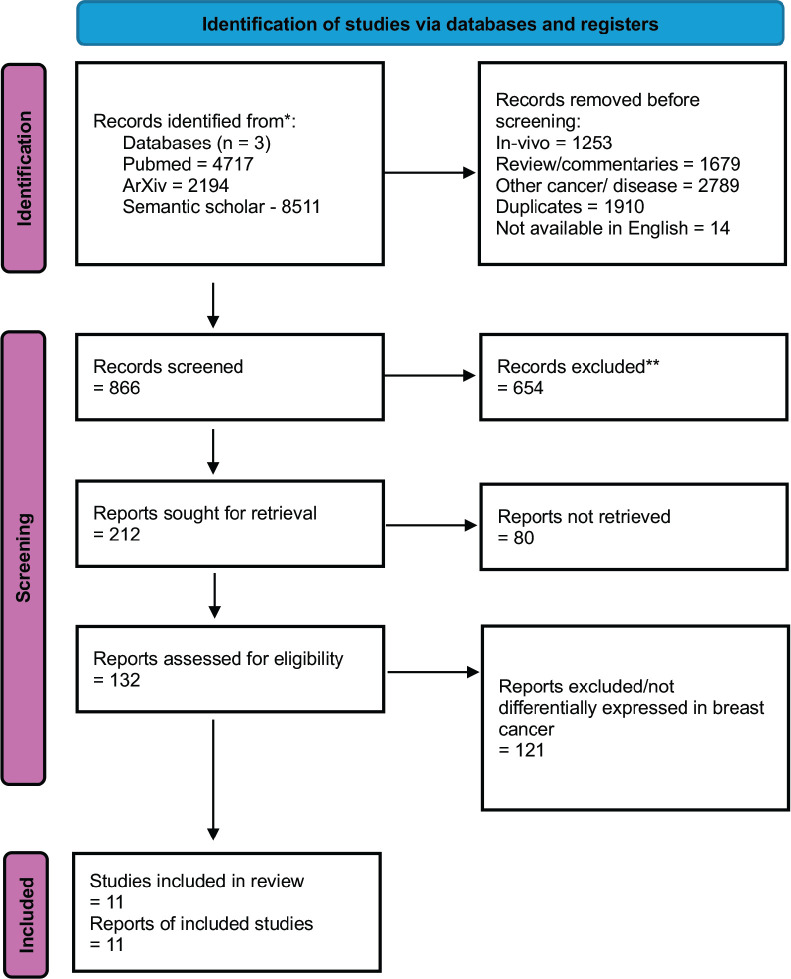
PRISMA flow diagram of study selection. Flow diagram illustrating the study selection process for the systematic review. Records were identified through database searching and screened using predefined inclusion criteria, followed by full-text assessment. A total of 11 studies were included for qualitative and quantitative synthesis.

Study selection followed a structured multistage workflow (Figure 2). Initial screening applied keyword-based filtering to titles, abstracts and indexed terms to remove clearly irrelevant records. Candidate studies then underwent computational relevance assessment using NLP-based classification aligned to predefined inclusion criteria. All shortlisted articles were subsequently reviewed manually to confirm eligibility based on methodological rigour and contextual relevance.

### Data extraction

Data extraction was performed independently by two investigators using a standardised framework, with discrepancies resolved by consensus. Extracted variables included study characteristics, patient demographics, molecular markers, detection methods, statistical outputs (including hazard ratios and fold changes) and reported clinical endpoints. Data were structured according to PICO elements to ensure consistency with the review objectives.

### Quantitative synthesis and statistical analysis

Quantitative synthesis was undertaken to estimate pooled effect sizes for molecular markers associated with MpBC diagnosis, progression and prognosis. Statistical significance was standardised at p < 0.05 and converted to Cohen’s d effect sizes to enable cross-study comparison.

**Figure 3.**
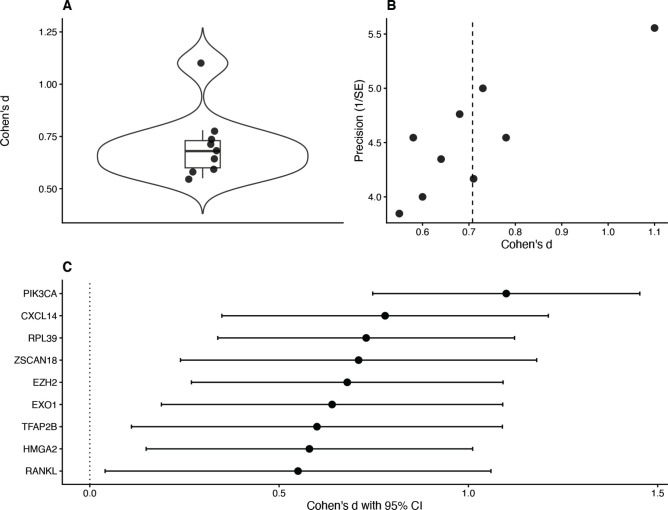
Effect-size distribution and meta-analytic assessment of MpBC-associated molecular markers. Distribution of standardised effect sizes (Cohen’s d) across candidate molecular markers identified from the systematic review.Funnel plot assessing publication bias, with the dashed line representing the mean effect size and Egger’s regression test used to evaluate asymmetry.Forest plot showing individual biomarker effect sizes with 95% confidence intervals. The dashed vertical line represents the pooled random-effects estimate.The pooled random-effects estimate was d = 0.74 [95% CI 0.59–0.88], supporting a moderate-to-large aggregate biomarker effect.

A random-effects meta-analysis was performed using the DerSimonian and Laird method. Between-study heterogeneity was assessed using the Q statistic, I² statistic and τ². Publication bias was evaluated using Egger’s regression test and visualised using funnel plots (Figure 3).

**Figure 4.**
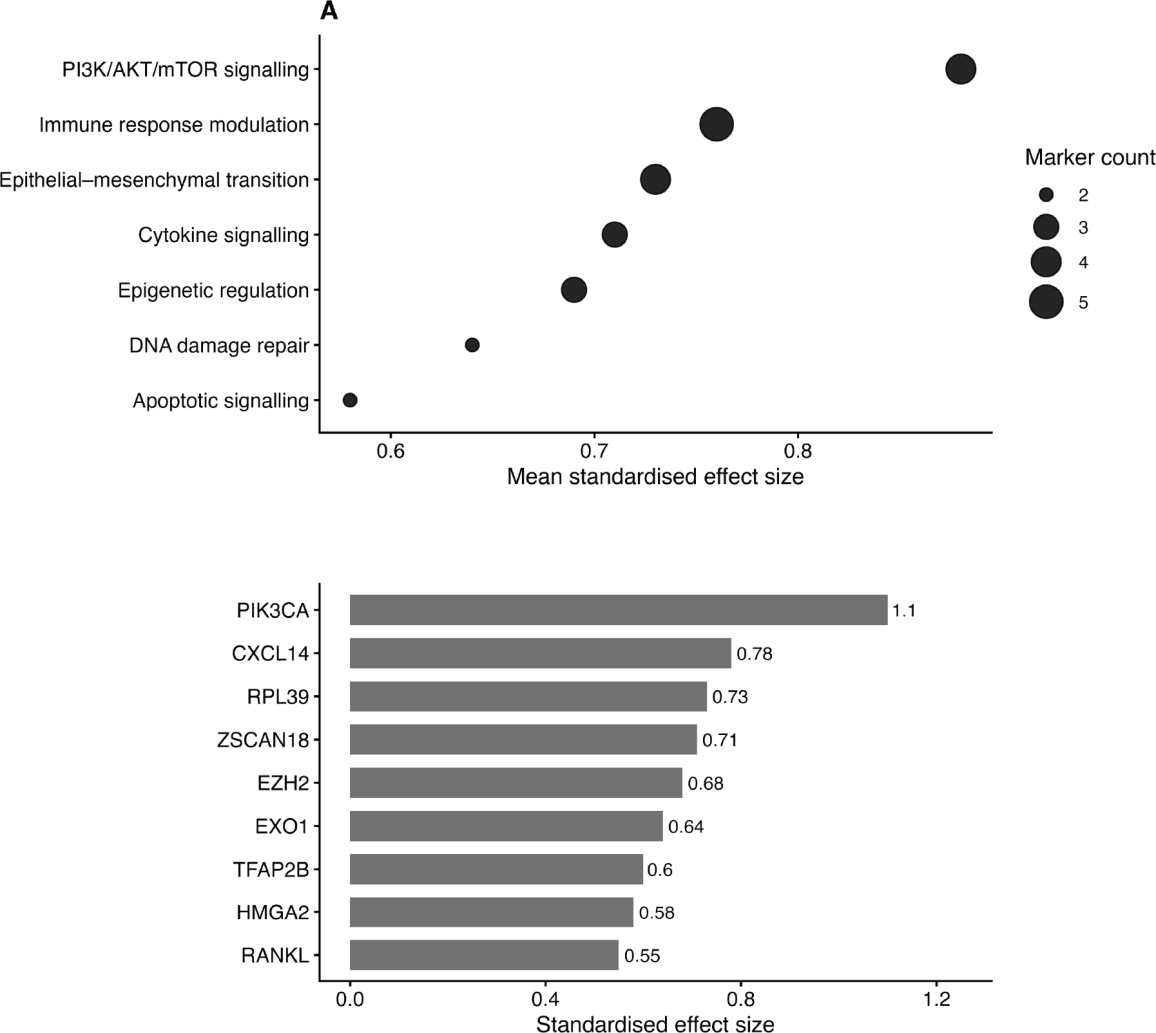
Pathway enrichment and biomarker prioritisation in metaplastic breast cancer. Dot plot summarising enriched biological pathways associated with candidate molecular markers, with point size reflecting the number of contributing biomarkers and position indicating mean standardised effect size (Cohen’s d).Ranking of candidate molecular markers by standardised effect size, highlighting PI3KCA, CXCL14 and RPL39 as high-priority markers.

Sensitivity analyses were conducted to assess the influence of individual studies on pooled estimates. Biomarkers were ranked based on effect size magnitude to support prioritisation and downstream interpretation (Figure 4).

All statistical analyses were performed in R (RStudio version 1.1.463), with data handling and visualisation implemented using packages including ggplot2, dplyr and meta. Code development and refinement were supported using Codex-assisted workflows to ensure reproducibility and transparency.

### Transcriptomic analysis

Transcriptomic analysis was performed using the GSE165407 dataset [12], comprising MpBC and TNBC/non-MpBC samples (n = 28; 8 MpBC, 20 non-MpBC). Differential gene expression analysis was conducted using the DESeq2 package in R, with thresholds defined as adjusted p < 0.05 and log₂ fold change > 1

Differentially expressed genes (DEGs) were visualised using volcano plots generated with ggplot2 (Figure 7a). Identified DEGs were cross-referenced with biomarkers extracted from the systematic review to generate a curated candidate gene set for downstream modelling.

### Machine learning modelling and validation

Supervised machine learning models were implemented to evaluate the predictive utility of candidate molecular markers. Two complementary approaches were used: a multi-layer perceptron (MLP) neural network and a boosted random forest (RF) classifier.

Data preprocessing included feature scaling using standardisation. The classification task was defined as binary (MpBC versus non-MpBC). Data were partitioned into training and test sets using a stratified split approach. Hyperparameter optimisation was performed using grid search where applicable.

Model performance was evaluated using accuracy, precision, recall and F1 score. Discriminative performance was assessed using receiver operating characteristic (ROC) analysis, with area under the curve (AUC) reported (Figure 9a). Classification outcomes were further summarised using confusion matrices (Figure 9b).

### Model interpretability

Model interpretability was assessed using SHapley Additive exPlanations (SHAP), enabling quantification of both global feature importance and local feature contributions to individual predictions.

SHAP summary plots and feature importance rankings were used to identify key gene-level drivers of model performance (Figure 9c). SHAP force plots were generated to illustrate the contribution of individual features to specific predictions, including directionality and magnitude of effect (Figure 9d).

## Results

### Systematic review and study characteristics

A total of 132 records were identified through database searching, of which 11 studies met the predefined inclusion criteria following multi-stage screening. The majority of exclusions occurred at the abstract screening stage due to lack of primary molecular data or insufficient relevance to MpBC. The study selection process is summarised in the PRISMA flow diagram (Figure 2).

The included studies encompassed a range of methodological approaches, including transcriptomic profiling, immunohistochemistry, multi-omic analyses and computational modelling. Collectively, these studies reported a diverse set of molecular markers supported by quantitative outputs such as differential expression, survival associations and odds ratios (Table 2). These markers formed the basis for subsequent quantitative synthesis and integrative analysis.

### Meta-analytic assessment of molecular markers

Quantitative synthesis demonstrated minimal between-study heterogeneity, with I² values approaching zero, indicating a high degree of consistency across reported biomarker effects. The pooled random-effects estimate was d = 0.74 (95% CI 0.59–0.88), consistent with a moderate-to-large aggregate effect size.

The distribution of effect sizes was relatively compact (Figure 3a), and funnel plot symmetry suggested minimal evidence of publication bias (Figure 3b). Forest plot analysis demonstrated narrower confidence intervals in larger studies, supporting the stability and robustness of pooled estimates (Figure 3c).

Among individual markers, RPL39 and CXCL14 demonstrated the largest effect sizes, while PI3KCA exhibited strong statistical robustness, reflected by consistently narrow confidence intervals across studies.

### Pathway enrichment and biomarker prioritisation

Pathway enrichment analysis identified several key biological processes associated with MpBC molecular markers, including PI3K/AKT/mTOR signalling, immune response modulation, cytokine signalling and epithelial–mesenchymal transition.

Functional enrichment network analysis (Figure 8) demonstrated dense connectivity between candidate biomarkers and enriched biological pathways, highlighting convergence across immune signalling, transcriptional regulation and cellular stress response processes. These findings support a model of MpBC biology characterised by coordinated dysregulation across multiple oncogenic pathways.

Effect size-based ranking (Figure 4b) identified PI3KCA, CXCL14 and RPL39 as the highest-priority markers, with additional contributions from ZSCAN18, EZH2, EXO1, TFAP2B and HMGA2. These markers collectively define a prioritised molecular signature for downstream validation.

### Clustering and research landscape analysis

**Figure 5.**
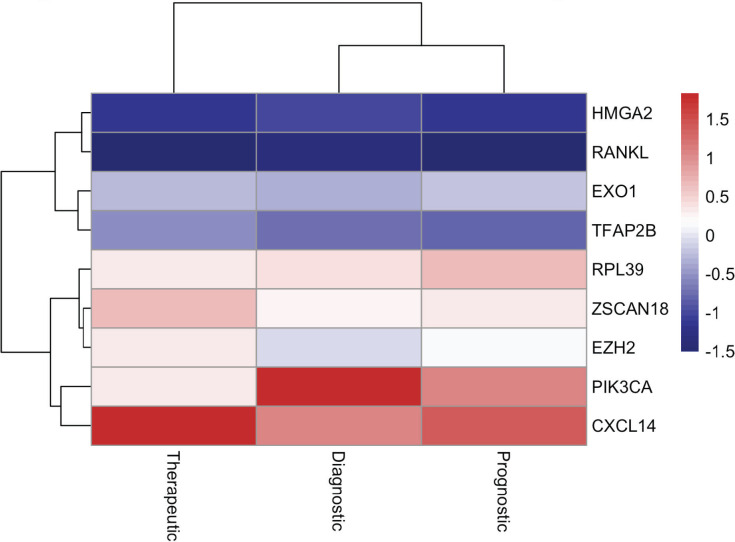
Clustering and functional classification of molecular markers in metaplastic breast cancer. Heatmap illustrating clustering of candidate molecular markers based on relative associations across diagnostic, prognostic and therapeutic domains. Values are scaled to Z-scores to enable comparison across markers. Hierarchical clustering highlights grouping of biomarkers with similar functional profiles, indicating convergence of molecular pathways relevant to metaplastic breast cancer biology.

Heatmap-based clustering demonstrated grouping of biomarkers according to diagnostic, prognostic and therapeutic relevance (Figure 5), suggesting functional convergence across distinct molecular classes. Hierarchical clustering revealed patterns consistent with shared biological pathways, including immune regulation and epigenetic control.

Temporal mapping indicated an increase in MpBC molecular research in recent years, although overall study numbers remain limited, reflecting the rarity of the disease.

**Figure 6.**
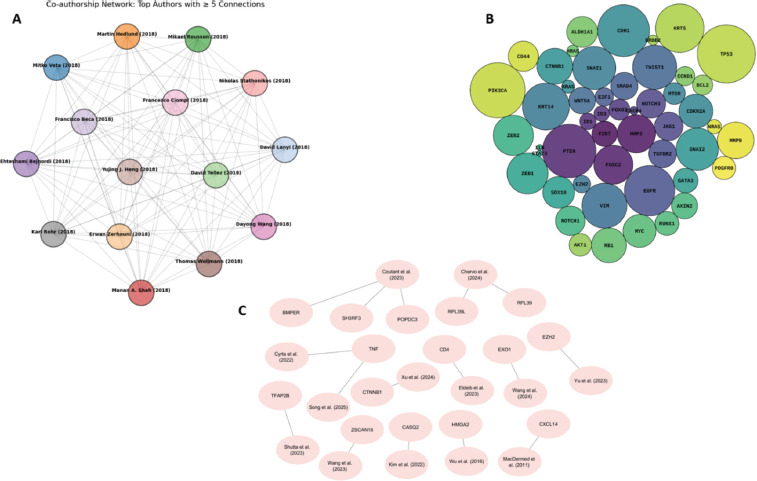
Co-authorship, molecular interaction and study–biomarker networks in metaplastic breast cancer research (a) Co-authorship network illustrating collaboration patterns among key contributors in metaplastic breast cancer biomarker studies, with nodes representing authors and edges indicating co-authorship relationships. (b) Network of molecular interactions highlighting key genes associated with metaplastic breast cancer, with node size reflecting relative importance or connectivity. (c) Study–biomarker network linking identified molecular markers to individual studies included in the systematic review.

Network-based analyses further revealed structured patterns of collaboration and molecular interaction. Co-authorship networks (Figure 6a) demonstrated emerging but fragmented research clusters, while gene–interaction and study–biomarker networks (Figure 6b–c) identified central nodes linking key biomarkers to multiple biological processes.

### Transcriptomic profiling and differential expression (Figure 7)

Transcriptomic analysis of the GSE165407 dataset (n = 28; 8 MpBC, 20 non-MpBC/TNBC) identified a subset of differentially expressed genes meeting predefined thresholds (adjusted p < 0.05, log₂ fold change > 1).

**Figure 7.**
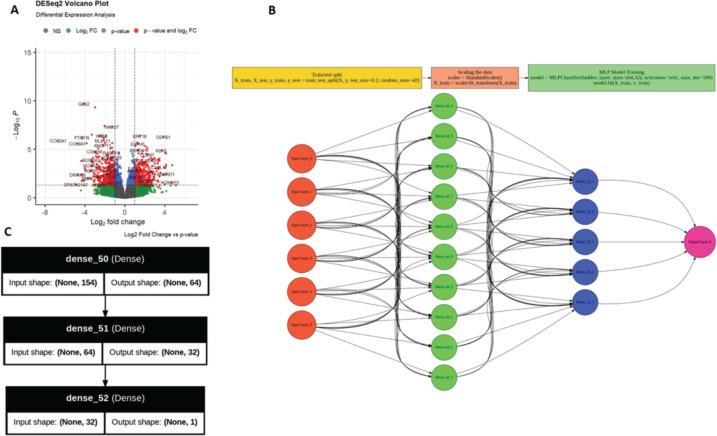
Transcriptomic differential expression and machine learning framework for metaplastic breast cancer classification (a) Volcano plot showing differentially expressed genes between MpBC and TNBC/non-MpBC samples, with thresholds defined by adjusted p < 0.05 and log₂ fold change. Selected genes are labelled to highlight key candidate markers. (b) Schematic representation of the machine learning workflow, including data splitting, feature scaling and multi-layer perceptron (MLP) model training. (c) Architecture of the neural network model, illustrating dense layer configuration and input–output dimensionality.

**Figure 8.**
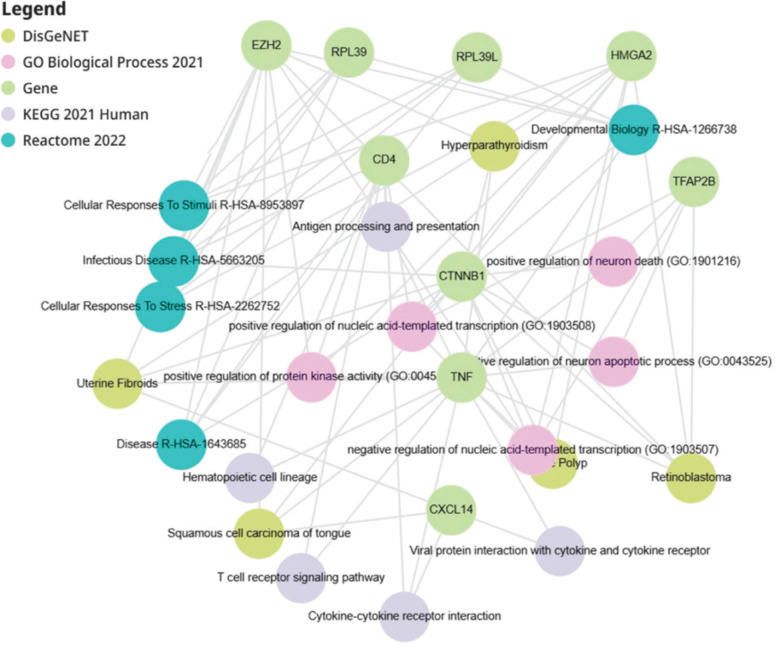
Functional enrichment network of candidate molecular markers in metaplastic breast cancer. Network representation of functional enrichment analysis linking candidate molecular markers to biological pathways and disease associations. Nodes represent genes and enriched terms derived from DisGeNET, Gene Ontology (Biological Process), KEGG and Reactome databases, with colours indicating annotation source. Edges denote functional associations between genes and enriched pathways, highlighting convergence on immune signalling, transcriptional regulation and cellular stress response pathways.

Volcano plot visualisation (Figure 7a) demonstrated clear separation of upregulated and downregulated genes, with several candidate markers overlapping with those identified in the systematic review. This concordance supports the validity of integrating literature-derived and data-driven approaches.

### Machine learning performance and validation (Figure 9)

**Figure 9.**
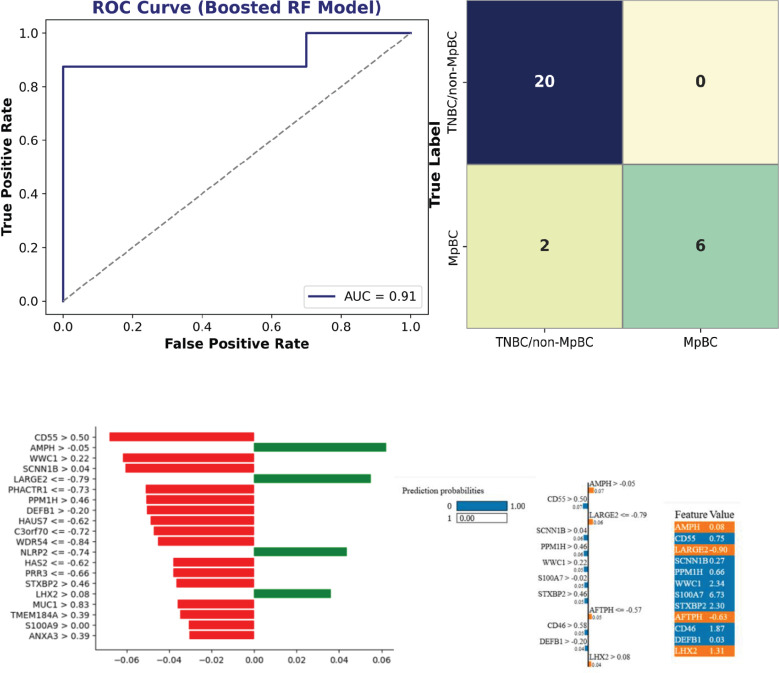
Machine learning model performance and SHAP-based interpretability for MpBC classification (a) Receiver operating characteristic (ROC) curve demonstrating model performance in distinguishing MpBC from non-MpBC samples, with an area under the curve (AUC) of 0.91. (b) Confusion matrix summarising classification performance, showing high specificity and sensitivity with minimal misclassification. (c) SHAP summary bar plot illustrating feature importance in the random forest model. Genes are ranked by their contribution to model output, with annotated thresholds indicating the values at which features most strongly influence classification (e.g., CD55 > 0.50, LARGE2 ≤ –0.79). Positive SHAP values (green) are associated with MpBC classification, whereas negative SHAP values (red) reflect contributions toward non-MpBC classification or protective effects. (d) SHAP force plot demonstrating individual prediction dynamics. The baseline represents the model’s expected output, while features shifting the prediction toward non-MpBC (class 0) are shown in blue and those driving prediction toward MpBC (class 1) are shown in orange. Gene-specific thresholds and corresponding expression values are displayed, highlighting the contribution of individual features to the final prediction.

The boosted random forest model demonstrated strong discriminative performance, achieving an area under the ROC curve of 0.91 (Figure 9a).

The confusion matrix (Figure 9b) indicated perfect specificity, with correct classification of all non-MpBC samples (20/20), and high sensitivity, with correct classification of 6 of 8 MpBC cases. Two MpBC samples were misclassified, with no false positives observed.

Feature importance analysis identified PI3KCA, RPL39 and EXO1 as key contributors to model performance, consistent with findings from both the systematic review and transcriptomic analysis.

### Model interpretability and SHAP analysis (Figure 9)

SHAP-based interpretability analysis demonstrated both global and local feature contributions to model predictions.

CD55 and LARGE2 emerged as influential features within the model, reflecting the broader transcriptomic feature space used in machine learning rather than exclusively literature-derived markers. Additional features, including AMPH, PPM1H, SCNN1B and WWC1, contributed variably to classification outcomes.

The directionality and magnitude of SHAP values were consistent with differential expression patterns, providing mechanistic insight into model decision-making and supporting biological plausibility (Figure 9c–d).

### Summary of prioritised molecular markers

Integration of findings from systematic review, meta-analysis, transcriptomic profiling and machine learning modelling yielded a prioritised set of molecular markers with potential diagnostic and prognostic relevance in MpBC (Table 3).

These markers converge on key biological processes, including epithelial–mesenchymal transition (HMGA2, RPL39), epigenetic regulation (EZH2), DNA damage response (EXO1) and immune signalling. Several markers, including PI3KCA and RPL39, demonstrated consistent prioritisation across all analytical layers, supporting their potential as robust candidates for further validation.

## Discussion

Metaplastic breast cancer (MpBC) remains a clinically aggressive and biologically complex malignancy, characterised by marked heterogeneity, intrinsic therapeutic resistance and poor outcomes despite standard treatment approaches [1,24,25]. A major limitation in the field has been the fragmentation of molecular evidence, with small, heterogeneous studies yielding inconsistent and often non-reproducible biomarker findings. In this context, the present study applies an integrative analytical framework combining systematic review, quantitative synthesis, transcriptomic validation and interpretable machine learning to identify and prioritise molecular markers with potential clinical relevance in MpBC.

A central observation is the convergence of independent analytical layers on a coherent set of biologically plausible markers. Genes including PI3KCA, CXCL14, RPL39, EZH2, HMGA2 and EXO1 demonstrated consistent prioritisation across systematic evidence synthesis, meta-analysis and transcriptomic profiling. The low heterogeneity observed in the meta-analysis and the compact distribution of effect sizes suggest that, despite the rarity of MpBC, a reproducible molecular signal can be identified when evidence is synthesised systematically. This addresses a key gap in the literature, where individual studies have often reported discordant findings without formal integration.

From a mechanistic perspective, the identified markers converge on pathways that are highly consistent with current understanding of MpBC biology. Dysregulation of the PI3K/AKT/mTOR axis is a recognised feature of aggressive breast cancer phenotypes and contributes to tumour progression, survival signalling and therapeutic resistance [27]. Similarly, the involvement of EZH2 supports a role for epigenetic reprogramming in driving tumour plasticity and lineage infidelity, while HMGA2 and RPL39 implicate epithelial–mesenchymal transition and cytoskeletal remodelling as key processes underlying invasion and metastasis. These findings collectively reinforce a model in which MpBC progression is driven by coordinated dysregulation of signalling, epigenetic control and cellular plasticity.

Immune-related signalling also emerged as a consistent theme. While the precise functional contribution of CXCL14 requires further elucidation, its association with immune-related pathways and its recurrence across analytical layers suggest a role within the tumour microenvironment. This is supported by broader evidence highlighting the importance of immune regulation and tumour–microenvironment interactions in aggressive breast cancer subtypes [28]. These observations raise the possibility that subsets of MpBC may exhibit immunologically relevant phenotypes, although further work is required to define their therapeutic implications.

An important distinction emerging from this study is the difference between biologically derived markers and those contributing to predictive model performance. While canonical markers such as PI3KCA and RPL39 were consistently prioritised across systematic and transcriptomic analyses, SHAP-based interpretability identified additional features, including CD55 and LARGE2, as influential within the classification model. These features reflect the broader transcriptomic feature space leveraged by machine learning and may not yet be fully characterised within the MpBC literature. Rather than representing discordance, this highlights the complementary roles of hypothesis-driven biology and data-driven modelling, and underscores the value of interpretability in bridging these domains.

From a clinical perspective, these findings have several important implications. First, the identification of a reproducible set of molecular markers provides a potential foundation for biomarker-driven stratification in MpBC, an area that remains poorly defined in current clinical practice. Second, the convergence of markers on key biological processes raises the possibility of integrating molecular profiling with established histological subtypes of MpBC, including squamous, spindle cell and mesenchymal variants. Such integration may enable more refined diagnostic classification and, ultimately, more tailored therapeutic approaches. Third, the identification of pathway-level dysregulation supports continued investigation into targeted therapeutic strategies, particularly those addressing signalling and epigenetic pathways. Stemming from this, the identification of EXO1 within the prioritised biomarker set further highlights the relevance of DNA repair pathways in MpBC, with emerging evidence suggesting that targeting exonuclease 1 (EXO1) and flap endonuclease 1 (FEN1) may enhance the efficacy of poly(ADP-ribose) polymerase (PARP) inhibition in triple-negative breast cancer contexts [29].

The translational trajectory of this work is being actively pursued. Validation of candidate markers in clinical tissue cohorts is planned through an Association of Breast Surgery grant application, incorporating immunohistochemical analysis in formalin-fixed paraffin-embedded specimens. In parallel, integration with radiomic analysis is being explored to identify imaging correlates of molecular signatures, with the aim of developing non-invasive biomarkers for diagnosis and treatment stratification. These approaches are particularly relevant in MpBC, where tumour heterogeneity and sampling limitations may constrain conventional diagnostic strategies. The findings of this study have also been presented at the British Association for Cancer Research conference (Edinburgh, June 2025), reflecting their relevance within the wider translational oncology community.

The strengths of this study lie in its integrative design. By combining systematic review, quantitative synthesis, transcriptomic validation and interpretable machine learning, this work addresses a fundamental limitation of prior MpBC research, namely the lack of reproducibility and cross-cohort validation. The concordance observed across analytical layers strengthens confidence in the identified biomarker set and supports its potential clinical relevance.

Several limitations should be acknowledged. The relatively small number of studies included in the systematic review reflects the rarity of MpBC and limits the breadth of available evidence. Similarly, the transcriptomic analysis is derived from a single dataset with a modest sample size, which constrains generalisability and may overestimate model performance. In addition, the current analysis focuses on gene-level associations and does not fully capture higher-order interactions within regulatory networks or the tumour microenvironment.

Future work should prioritise validation in larger, multi-centre cohorts and expand to incorporate additional multi-omic layers, including proteomic and epigenomic profiling. The development of combinatorial biomarker panels is likely to be essential to capture the biological complexity of MpBC. Ultimately, translation into clinical practice will require prospective validation and incorporation into biomarker-driven clinical trial designs.

## Conclusion

In summary, this study provides an integrated and reproducible framework for molecular marker identification in MpBC, demonstrating convergence across systematic evidence, transcriptomic data and machine learning models. By resolving inconsistencies in the existing literature and identifying biologically coherent molecular signatures, these findings provide a foundation for future translational research and potential clinical application in this challenging disease.

## Future directions

Building on this foundation, future work from our group will investigate context-specific biomarker panels optimised for distinct MpBC histologies and clinical applications, including therapeutic versus diagnostic combinations. Experimental validation of the identified gene signatures in larger, multicentre cohorts is critical to confirm reproducibility and generalisability. In parallel, broader bioinformatic exploration across additional high-throughput datasets will be undertaken to refine our molecular framework. Collectively, these efforts aim to support the integration of predictive molecular tools into routine clinical care for MpBC and to lay the groundwork for prospective, biomarkerin-formed therapeutic trials.

## Author contributions

Study conceptualisation - SS. Supervision - SS. Data curation - JA, SS. Formal analysis - JA, SS. Visualisation - JA, SS. Writing - original draft - JA. Writing, review & editing - SP, NT, MV, JS, SS.

## Conflicts of interest

The authors declare no conflicts of interest.

## Funding

This review has no specific/external funding.

## Data availability

The datasets generated and analysed during the current study are available from the corresponding author on reasonable request. All data have been de-identified to protect patient privacy and in accordance with applicable ethical guidelines.

## Registration number

CRD420251019399, PROSPERO 2025.

## Figures and Tables

**Figure 1.**
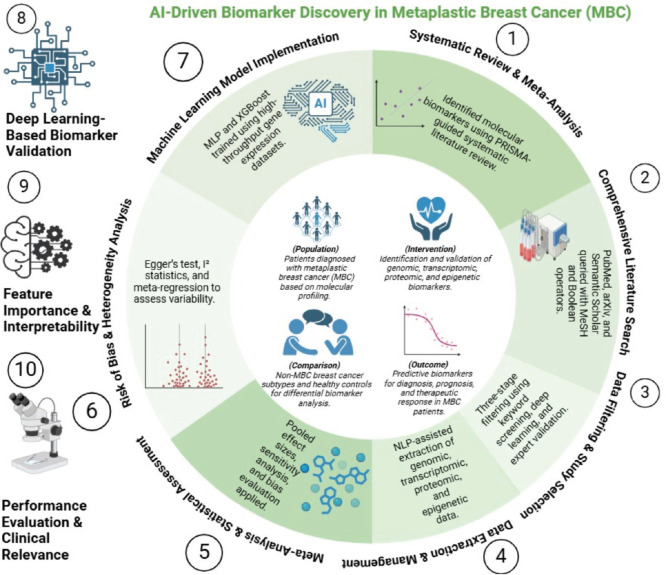
Integrative pipeline for molecular marker identification in metaplastic breast cancer. Schematic overview of the analytical workflow integrating systematic review, transcriptomic profiling and machine learning. Candidate markers identified from the literature were cross-referenced with differentially expressed genes from the GSE165407 dataset. Supervised models and downstream pathway analyses were applied to prioritise biologically and clinically relevant molecular signatures.

**Table 1.** Structured search strategy for identification of molecular markers in metaplastic breast cancer. Search queries were developed using Medical Subject Headings (MeSH) terms and free-text keywords, combined with Boolean operators to maximise sensitivity and specificity. Categories reflect major domains of biomarker research, including genomic, proteomic, liquid biopsy and artificial intelligence–driven approaches. Queries were adapted across databases (PubMed, arXiv and Semantic Scholar) to ensure comprehensive retrieval of relevant studies.

Category	Search Strategy
General biomarkers	(‘Molecular Biomarkers’[MeSH] OR ‘Genetic Markers’[MeSH] OR ‘Tumor Markers, Biological’[MeSH] OR ‘Biomarkers, Tumor’[MeSH]) AND (‘Metaplastic Breast Cancer’[MeSH] OR ‘Metaplastic Breast Neoplasms’) AND (‘Diagnosis’[MeSH] OR ‘Prognosis’[MeSH] OR ‘Prediction’)
Genomic / epigenetic markers	(‘Genetic Markers’[MeSH] OR ‘Epigenomics’[MeSH] OR ‘DNA Methylation’[MeSH] OR ‘Gene Expression Profiling’[MeSH]) AND (‘Metaplastic Breast Cancer’) AND (‘Diagnosis’ OR ‘Prognosis’ OR ‘Risk Factors’)
Proteomic markers	(‘Proteomics’[MeSH] OR ‘Protein Biomarkers’ OR ‘Oncoproteins’[MeSH] OR ‘Cytokines’[MeSH]) AND (‘Metaplastic Breast Cancer’) AND (‘Prediction’ OR ‘Prognostic Value’)
Liquid biopsy markers	(‘Circulating Tumor Cells’[MeSH] OR ‘Exosomes’[MeSH] OR ‘Cell-Free Nucleic Acids’[MeSH] OR ‘ctDNA’) AND (‘Metaplastic Breast Cancer’) AND (‘Early Detection’ OR ‘Survival Analysis’)
AI / ML approaches	(‘Artificial Intelligence’[MeSH] OR ‘Machine Learning’[MeSH] OR ‘Deep Learning’) AND (‘Molecular Biomarkers’ OR ‘Genomic Markers’) AND (‘Metaplastic Breast Cancer’) AND (‘Prediction’ OR ‘Prognosis’)
Systematic reviews / meta-analyses	(‘Biomarkers’ OR ‘Prognostic Biomarkers’) AND (‘Metaplastic Breast Cancer’) AND (‘Systematic Review’ OR ‘Meta-Analysis’)

**Table 2.** Summary of included studies reporting molecular markers in metaplastic breast cancer. Included studies are summarised by population, methodological approach, comparator group, principal finding and molecular marker. Studies encompass transcriptomic, proteomic, immunohistochemical, computational and experimental approaches. Markers not present in the GSE165407 dataset, including BMPER, POPDC3 and SH3RF3, were not included in downstream machine learning analyses. The original proof table included these 11 studies and associated markers.

Study	Population	Methodology	Comparator	Key findings	Molecular marker(s)
Coutant et al [13]	TNBC cohort including MpBC cases	Spatial transcriptomics, pathological annotation, differential expression and copy number analysis	TCGA and CCLE datasets	Identified markers of high-plasticity breast cancer with specificity for MpBC-like tumour regions	BMPER, POPDC3, SH3RF3
Eldeib et al [14]	63 patients with invasive breast carcinoma	Immunohistochemical assessment of PI3KCA, CD4 and CD8 tumour-infiltrating lymphocytes	Molecular subtype comparison	PI3KCA positivity and altered CD4/CD8 immune balance were associated with adverse clinicopathological features	PI3KCA
Yu et al [15]	HER2-positive breast cancer cohort	Immunohistochemical assessment of EZH2 expression and phosphorylation status	Low EZH2 expression cases	Nuclear EZH2 expression and site-specific phosphorylation were associated with tumour invasiveness and therapeutic resistance	EZH2
Wang et al [16]	Breast cancer tissue and public expression/methylation datasets	In silico expression profiling and DNA methylation analysis	Normal breast tissue	ZSCAN18 was downregulated in breast cancer and promoter hypermethylation was implicated as a mechanism of suppression	ZSCAN18
Cyrta et al [17]	27 breast carcinomas with osteoclast-like giant cells	RNA sequencing, histopathological review, genomic profiling and immunohistochemistry	Invasive carcinoma NST without osteoclast-like giant cells	RANKL-associated signalling and osteoclast-related pathways were enriched, suggesting a tumour microenvironment-driven phenotype	TNFSF11/RANKL
Kim et al [18]	TCGA breast cancer dataset and experimental breast cancer models	Gene expression analysis, in vitro overexpression studies and in vivo modelling	Normal breast tissue and control cell lines	CASQ2 overexpression promoted EMT-associated features, tumour progression and metastasis	CASQ2
Song et al [19]	Breast cancer samples from TCGA and GTEx	Pan-cancer bioinformatic analysis, survival modelling and immune infiltration analysis	Tumour versus normal tissue; EXO1-high versus EXO1-low groups	EXO1 was overexpressed in breast cancer and associated with adverse survival, advanced stage and immune infiltration	EXO1
Weighill et al [20]	TCGA breast cancer multi-omic data	Gaussian graphical modelling for multi-omic network inference	Existing network inference approaches	Multi-omic regulatory network analysis identified TFAP2B as a candidate marker associated with basal-like breast cancer biology	TFAP2B
Dave et al [5]	40 patients with MpBC, supported by cell line and PDX models	Genomic analysis, in vitro functional assays and in vivo iNOS inhibition	Vehicle and docetaxel-treated controls	RPL39 alteration was linked to iNOS signalling, tumour growth, migration and chemoresistance	RPL39
Wu et al [21]	273 training and 310 validation breast cancer specimens	Immunohistochemistry and survival analysis	Low versus high HMGA2 expression	Elevated HMGA2 expression was associated with tumour aggressiveness, metaplastic phenotype and poor outcome	HMGA2
MacDermed et al [22]	Triple-negative breast cancer progression model	Gene expression profiling, qPCR, ELISA and xenograft knockdown experiments	TES-1 versus TES-2b models	CXCL14 promoted tumour growth and was implicated in inflammatory, stem-like and metastatic breast cancer progression	CXCL14

**Table 3.** Key molecular markers in metaplastic breast cancer and their functional and clinical relevance. Selected biomarkers identified through integrative analysis are summarised with their associated biological functions, clinical relevance and classification as diagnostic, prognostic or therapeutic markers. Functional roles are derived from reported molecular mechanisms, including pathways related to epithelial–mesenchymal transition, immune modulation and epigenetic regulation. Clinical relevance reflects associations with tumour progression, treatment response and patient outcomes.

Biomarker	Functional Role	Clinical Relevance	Biomarker Type	Reference
EXO1	DNA repair and replication stress response	Associated with poor survival and tumour progression	Prognostic	Song et al [19]
TNFSF11 (RANKL)	Osteoclast signalling and tumour microenvironment modulation	Linked to aggressive tumour phenotype and inflammatory signalling	Prognostic / Therapeutic	Morgan et al [26]
HMGA2	Chromatin remodelling and epithelial–mesenchymal transition	Correlates with tumour aggressiveness and adverse outcomes	Diagnostic / Prognostic	Wu et al [21]; Kalaw et al [4]
EZH2	Epigenetic regulation via PRC2-mediated gene silencing	Associated with metaplastic progression and therapeutic resistance	Diagnostic / Prognostic / Therapeutic	Morgan et al [26]
PI3KCA	PI3K/AKT/mTOR pathway activation	Frequently altered in MpBC; potential target for pathway inhibition	Prognostic / Therapeutic	Reddy et al., 2020 [27]
CXCL14	Immune modulation and tumour microenvironment signalling	Associated with immune infiltration and metastatic potential	Prognostic / Therapeutic	Gibbs et al., 2022 [28]
RPL39	Ribosomal protein regulating iNOS signalling	Linked to aggressive phenotype and chemoresistance	Prognostic	Dave et al [5]
TFAP2B	Transcriptional regulation in tumour progression	Implicated in basal-like and metaplastic tumour biology	Diagnostic	Zhang et al [6]
